# Nano-scale evidence for osteocyte network integration across bone remodeling interfaces in human bone revealed by synchrotron nanoCT

**DOI:** 10.1016/j.mtbio.2026.102813

**Published:** 2026-01-16

**Authors:** Sophie Anuth, Emely Bortel, Julie Villanova, Jussi-Petteri Suuronen, Sven Geissler, Amaia Cipitria, Peter Fratzl, Tobias Fretwurst, Katja Nelson, Susanne Nahles, Bernhard Hesse

**Affiliations:** a Xploraytion GmbH, Invalidenstrasse 34, 10115, Berlin, Germany; b Group of Bioengineering in Regeneration and Cancer, Biogipuzkoa Health Research Institute, San Sebastián, Spain; c Department of Cell Biology and Histology, Faculty of Medicine and Nursing, University of the Basque Country UPV/EHU, Leioa, Spain; d ESRF – European Synchrotron Facility, Avenue des Martyrs 71, 38043, Grenoble, France; e Berlin Institute of Health Center for Regenerative Therapies, Charité - Universitätsmedizin Berlin, Berlin, Germany; f IKERBASQUE, Basque Foundation for Science, Bilbao, Spain; g Department of Biomaterials, Max Planck Institute of Colloids and Interfaces, Max Planck Society, Am Mühlenberg 1 OT Golm, 14476, Potsdam, Germany; h Department of Oral and Craniomaxillofacial Surgery/Translational Implantology, Center for Dental Medicine, Medical Center, Faculty of Medicine, University of Freiburg, Hugstetter Straße 55, Freiburg, 79106, Germany; i Department of Craniomaxillofacial Surgery, Charité, CVK, Augustenburger Platz 1, Berlin, 13353, Germany

**Keywords:** Osteocyte lacunar-canalicular network (OLCN), Canalicular connectivity, Cement lines, Bone remodeling, Synchrotron nano computed tomography, Bone tissue nano-architecture

## Abstract

Bone remodeling is a highly regulated, hierarchical process critical for maintaining structural integrity and mineral homeostasis. At the nano-scale, the osteocytes orchestrate mechanosensing, signaling, and nutrient transport across the mineralized matrix utilizing their extensive network of cell dendrites. The lacunar-canalicular network (OLCN) houses the cellular components within the matrix. How this network integrates across bone regions formed during different remodeling cycles remains unresolved. How the cellular network is connected across interfaces between different remodeling regions or cement lines is the focus of this exploration: is the network integration merely stochastical occurrences or result of a cued, directed formation process?

Using synchrotron-based nano computed tomography (nano-CT), we analyze human bone samples of 35 different patients with sub-micron resolution to characterize canalicular structures around cement lines. The results show the network's ability and affinity to integrate, and the strong influence of local tissue conditions on the degree of integration. We novelly include the structural analysis of canalicular network architecture to interpret underlying formation processes. Besides 'cross-generational' canalicular connections, we identify previously overlooked canalicular loops in newly formed bone near cement lines and interpret these as morphological indicators of a directed, adaptive search for reconnection. The study suggests a mechanism combining random outgrowth and directed progression influenced by local cues.

We propose a 'cross-generational' OLCN: a deliberately integrated network that enhances tissue connectivity, functional resilience, and osteocyte survival across temporal remodeling stages. These findings advance the understanding of bone network complexity and introduce canalicular looping as a nano-structural signature of directed formation in bone network architecture.

## Graphical abstract

**Figure:**
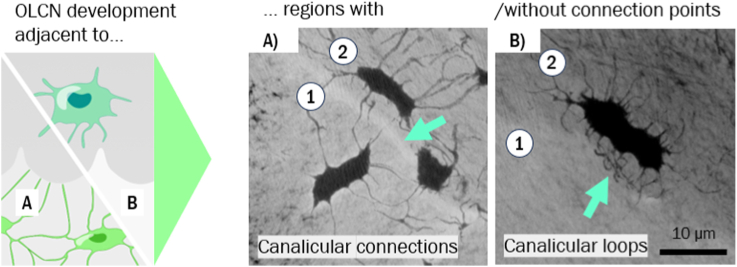
Network development/ formation of canalicular patterns and resulting network integrity across cement lines is shown as a response to existence of possible connection points within the adjacent pre-existing mineralized matrix. A) Case one – formation of “cross-generational” connections - given the presence of possible connection points and B) case two – formation of loops – in absence of connection points within the pre-existing matrix are visualized with minimum intensity projections over 10 μm. Locally younger (re)modeling regions is indicated with (1), locally older region with (2), the cement line interface with a turquois arrow.

## 1 Introduction

The human organism relies on the skeletal system for stability, locomotion, protection of organs, and as a mineral reservoir [1]. Designed to fulfill these diverse functions, bone is a highly functional composite material built and maintained by a well-organized interplay of cellular activity and continuous remodeling, guaranteeing structural integrity and adaptation to changing mechanical demands [2,3]. The remodeling process of mature human bone involves two spatially coupled activities: resorption of the mineralized matrix by osteoclasts, followed by deposition and mineralization of new bone tissue by osteoblasts. This procedure is orchestrated by osteocytes as the central regulators [2,4,5]. Osteocytes are the most abundant bone cells, with a density of tens of thousands per mm^3^ [6,7], embedded in lacunae within the mineralized bone matrix and interconnected through cell dendrites [8,9]. These dendrites extend through nano-scale channels, canaliculi, which form the intricate osteocyte lacunar-canalicular network (OLCN). The cellular network is fundamental for transport and exchange of nutrients and minerals and the overall maintenance of tissue homeostasis [[10], [11], [12]]. It is also key to the osteocytes’ mechano-sensation and signal transmission and thereby their ability to direct the remodeling process [13,14].

Bone remodeling creates a patchwork of regions of different local tissue ages constituting the mineralized matrix. Tissue regions formed in different remodeling cycles are characteristically separated by hyper-mineralized structures known as cement lines. These interfaces of a few micrometer thickness are formed after tissue resorption, prior to the formation of the new matrix and the inherent network [15,15]. The hyper-mineralized structures have once been considered impenetrable [16] or infrequently crossed [17]. This characteristic would entail a discontinuity of the OLCN and significantly limit network functionality. The integration of the network therefore seems intuitively favorable, and indeed, more recent research reports canaliculi crossing the cement line, integrating the networks of adjacent regions [11,18].

Aided by continuous advances in nano imaging, recent research has focused on analyzing the characteristics of the lacunar-canalicular network [[19], [20], [21]], and its involvement in bone remodeling [22,23].Phase contrast synchrotron X-ray nano-tomography (SR nano-CT) is particularly suited for the complex task of imaging the bone ultrastructure down to the OLCN. Compared to other techniques, SR nano-CT uniquely combines nanometer resolution with a large field of view (>10^6^ μm^3^ [24]) and relatively fast acquisition times. It enables a comprehensive analysis of large segments of the intricate nano-scale network [19]. Importantly, this can be achieved non-destructively, without the need for demineralization or tissue sectioning, enabling the visualization of a network unimpaired by sample preparation.

Compared to confocal microscopy, an alternative imaging method frequently used to visualize the OLCN [25], SR nano-CT is penetration-depth independent, and provides isotropic nanoscale resolution throughout intact mineralized bone. While the fluorescence labeling used for confocal imaging offers advantages in the segmentation of the OLCN, the characterization of the network architecture is limited by imaging depth [26].

The unique characteristics of SR nano-CT imaging are crucial for gaining representative insights on the three-dimensional configuration of the vast network structure spanning the entire organ. Additionally, the use of phase contrast in synchrotron-based tomography facilitates the direct correlation of voxel intensity with physical density of the sample, providing a measure of the degree of mineralization in the scanned region based on gray value (GV) assessment [27]. The mineralization of the tissue is a continuous process, resulting in a correlation between local tissue age and degree of mineralization [28,29]. This allows the relative interpretation of the local tissue age of different (re)modeling regions and the localization of (re)modeling events. Younger, less mineralized matrix regions display lower gray levels in the CT images which increase with mineralization and local tissue age over time. Accordingly, the hyper-mineralized cement lines feature locally increased gray values [15]. Resorption creates a distinct interface between mineralized and soft tissue, where osteoclasts actively erode the matrix. This interface presents a sharp contrast and wave-like morphology, mirroring the pattern of aligned resorption pits [30]. The resorption front topology also determines that of the cement line and is thus preserved in the mature matrix. Mineralization results in a gray value gradient at the interface between the existing mineralized matrix and soft tissue. Active remodeling can visually be distinguished by resorption fronts and layers of mineralizing tissue in direct proximity [3]. Based on these considerations correlations between network architecture and local tissue age, as well as interpretations of matrix features in the context of matrix constitution and (re)modeling can be drawn.

This study utilizes this, building upon the analysis of SR nanoCT data of bone (re)modeling, and explores the role of the canalicular network architecture in these events. The main question is, whether OLCN integrity and canalicular connections across cement lines are merely stochastically formed or the result of a systemic mechanism. In this context, patterns of the canalicular architecture are considered as indicators of the underlying construction process.

The OLCN and canalicular patterns are examined in areas of active and completed (re)modeling. Regions of interest specifically include: (i) sites of active osteoclastic bone resorption, (ii) areas of early osteoblastic tissue mineralization, and (iii) regions where both processes occur simultaneously along the surface or within a basic multicellular unit (BMU). Additionally, (iv) areas in proximity to cement lines are analyzed as they allow the assessment of the concluded (re)modeling process. (In the following study both formation and remodeling processes are considered. For the sake of readability, we will stick to the term “remodeling” in the explanations.)

This study analyses 85 SR phase contrast nano-CT datasets (voxel size: 50 nm) from 49 human bone samples across various anatomical sites, including alveolar bone, femur, fibula, and the iliac crest. The sample cohort includes both healthy and pathological conditions, spanning donors of different ages and sexes.

## 2 Results

### 2.1 Bone (re)modeling events

**Fig. 1:**
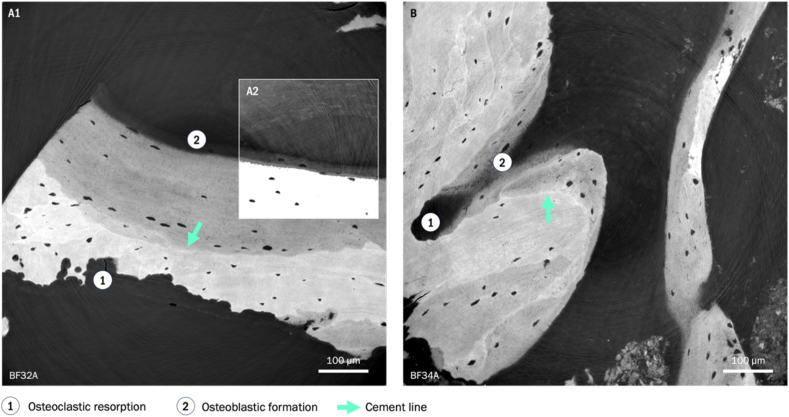
Individual SR CT cross-sections (voxel size: 240 nm) showing sites of bone remodeling activity: A1) formation along the upper and resorption along the lower interface in two distinct remodeling units separated by the cement line*, difference in local tissue age is visible in different gray values of the upper (darker = younger) and lower region (lighter = older), A2) shows the same slice with adjusted contrast revealing the mineral brim and settling osteocytes in formation; B) shows Haversian remodeling in a BMU.*

To perform an analysis of the canalicular network architecture in remodeling, remodeling events were localized based on distinct visual features of the tomographic image data. These include resorption, formation, and regions around cement lines displaying the concluded remodeling activity. Individual remodeling regions and the separating cement lines were defined (Fig. 1).

### 2.2 Morphological analysis of OLCN patterns in (re)modeling

The nano-scale resolution of the SR nano-CT facilitates the assessment of canalicular patterns in the context of remodeling events. After localizing remodeling sites, we identified reoccurring morphologies of the OLCN architecture.

**Fig. 2:**
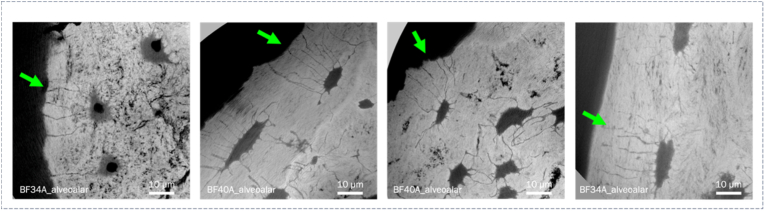
Minimum intensity projections over 10 μm showing orthogonal canalicular orientation towards resorption front.

**Fig. 3:**
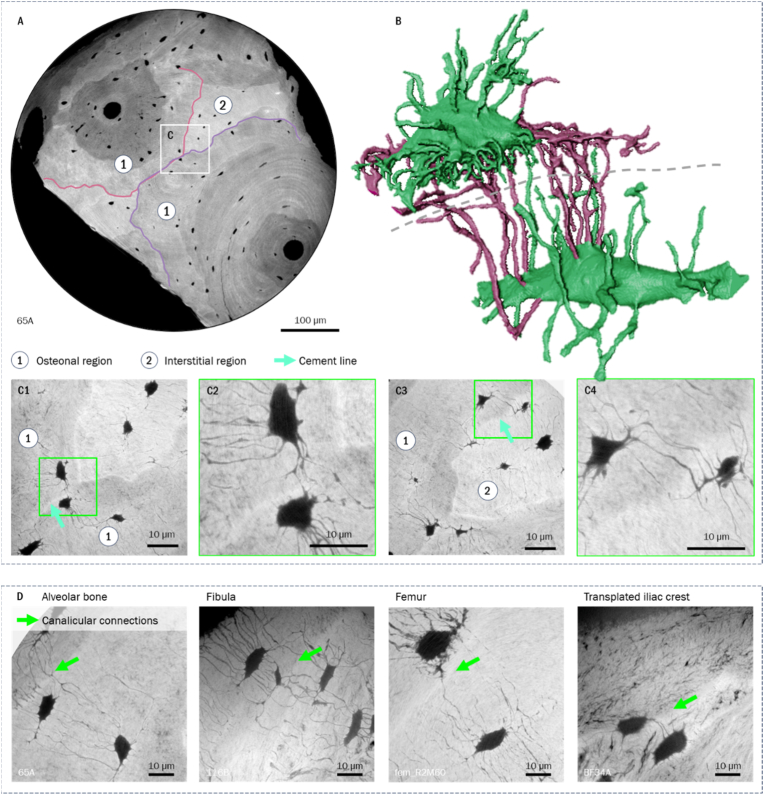
A) Cross-section of overview scan at 240 nm voxel size indicating osteonal and interstitial tissue regions; B) exemplary 3D rendering of to *'cross-generationally' connected lacunae (cement line position indicated by dashed line, crossing canaliculi highlighted in red). C*1) and C3) show minimum intensity projections over 10 μm of the nanoCT scan at the in A) highlighted position, C2) and C4) zoom-ins showing canalicular connections; in C1, 2) the shown connections are between the osteonal regions, in C3, 4) between osteon and interstitial tissue. In D) ‘cross-generational’ connections are shown within different bone/sample types in minimum intensity projections over 10 μm. (For interpretation of the references to colour in this figure legend, the reader is referred to the Web version of this article.)

In regions of osteoclast activity, canaliculi displayed a predominantly perpendicular orientation to the resorption surface (Fig. 2). Withing the locally younger remodeling units, we identified two distinct canalicular network patterns: ‘cross-generational’ connections and canalicular loops. We observed ‘cross-generational’ canalicular connections within different remodeling regions (resorption and formation) in most analyzed datasets. Image volumes depicting early stages of mineralization revealed canalicular connections between lacunae within the old and the newly formed matrix. Their long-term structural persistence could be shown in scans of regions in proximity to cement lines within the fully mineralized matrix. They were found linking osteonal and interstitial regions, as well as different osteons (Fig. 3).

**Fig. 4:**
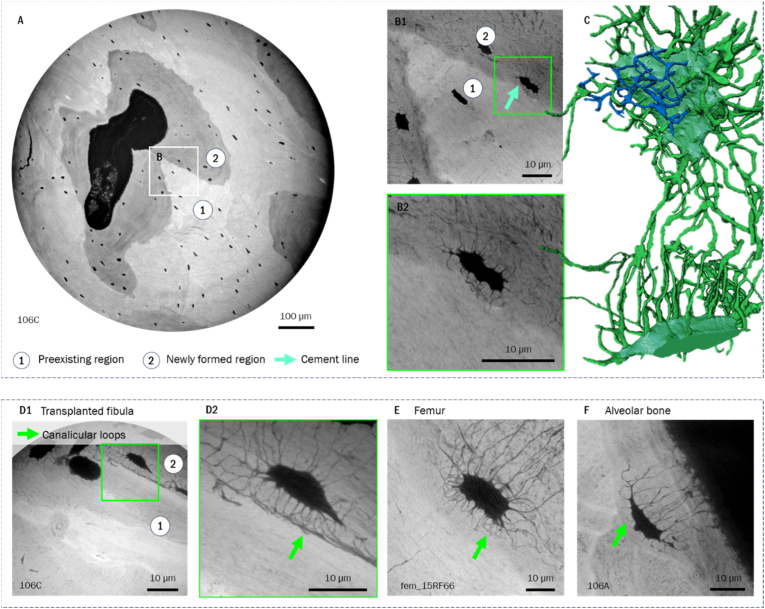
A) Cross-section *of overview scan at* 240 nm *voxel size indicating preexisting and newly formed tissue regions; B1) shows a minimum intensity projection over* 10 μm *of the nanoCT scan at the in A)* highlighted position, B2) zoom-in showing canalicular *loops; C) shows an exemplary 3D rendering of two lacunae neighboring the cement line (canalicular loops highlighted in blue). I*n *D) and E) canalicular loops are shown within different bone/sample types in minimum intensity projections over* 10 μm*; with D2) as a zoom-in at the position highlighted in D1)*. (For interpretation of the references to colour in this figure legend, the reader is referred to the Web version of this article.)

Additionally, facing cement lines bordering regions of low network density or completely void thereof, we observed canalicular loops These loops are only found in locally younger tissue regions (Fig. 4). Both patterns could also be localized within remodeling units in direct proximity to one another.

Both patterns were confirmed in all analyzed sample types independent of donor age or sex. Visualizations of canalicular connections and loops in one example location for each sample are included in the supplementary information, **Figure SI1**.

### 2.3 Quantification of canalicular connections and canalicular porosity

**Fig. 5:**
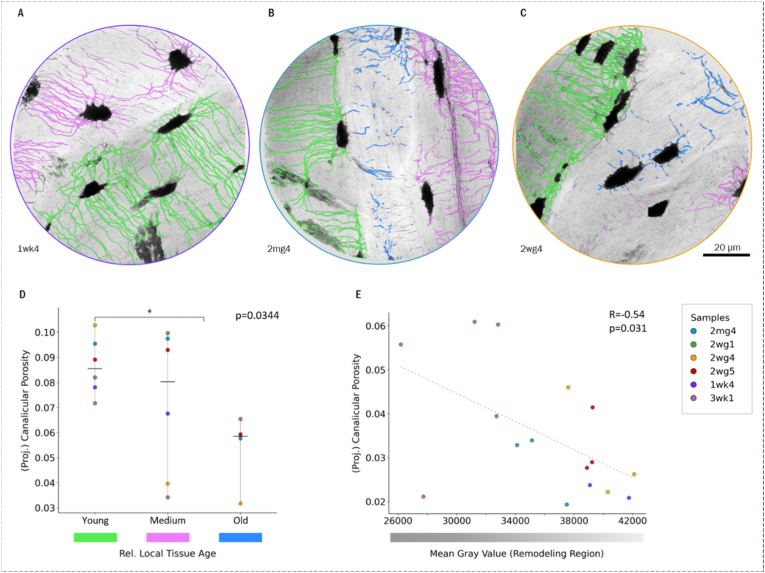
A-C) Show the regional canalicular masks within 3 exemplary samples (green: canaliculi within locally youngest region, pink: medium, blue: oldest region); the different local tissue age can be assessed from the regional gray scale differences. D) shows the results of the (projected) canalicular porosity (calculated per projection) over relative local tissue age of the individual remodeling regions per sample; in E) projected canalicular porosity distribution over regional mean gray value is shown. Patient age per sample: 2mg4 - 42 y, 2wg1 - 68 y, 2wg4 - 47 y, 2wg5 - 42 y 1wk4 - 75 y, 3wk1 - 72 y. (For interpretation of the references to colour in this figure legend, the reader is referred to the Web version of this article.)

**Fig. 6:**
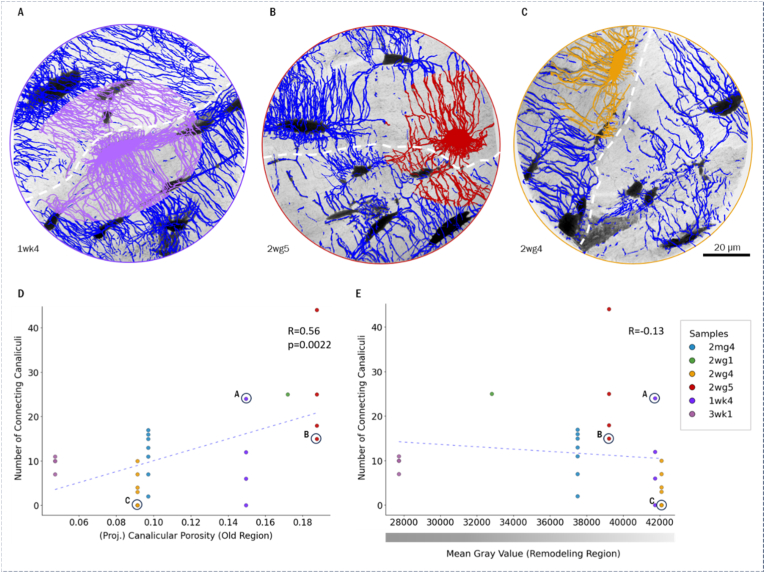
A -C) Show the segmented canaliculi crossing cement lines per lacuna for 3 exemplary lacunae. *D*) shows the results of the number of canalicular connections per lacuna (within locally younger region) over projected canalicular porosity of the neighboring (older) region*, E) number of canalicular connections* per *lacuna over mean gray value of the neighboring region;*, data points of exemplarily visualized lacunae are highlighted. Patient age per sample: 2mg4 - 42 y, 2wg1 - 68 y, 2wg4 - 47 y, 2wg5 - 42 y 1wk4 - 75 y, 3wk1 - 72 y.

Canalicular connections across cement lines are found in at least one location in 70 % (N_c_ = 60) of all analyzed image volumes and canalicular loops in 75 % (N_l_ = 64). Quantitative analysis of relative local tissue age and canalicular porosity was performed based on minimum intensity projections on 6 alveolar bone sample data sets. The results per region are listed in **Table SI2**. For each sample, regions are categorized by local tissue age from youngest to oldest and the corresponding (projected) canalicular porosities assessed relative to each other. The median (projected) canalicular porosity within the younger regions was found to be significantly higher (p = 0.034) compared to the median over the older regions. A significant negative correlation for the canalicular density versus regional mean GV of the mineralized matrix normalized to the mean GV of the lacunae within the region was also found (p = 0.031) (Fig. 5). The analysis of the number of canalicular connections per lacuna showed a significant dependency on the degree of mineralization of the neighboring old tissue region (R = 0.56, p = 0.002). It is also noticeable that the patient's age appears to be a lesser impact as the samples from younger patients are spread throughout the range (Fig. 6). The results per lacuna per sample are listed in **Tabel SI.3**.

## 3 Discussion

### 3.1 Directed formation of ‘cross-generational’ connections

**Fig. 7:**
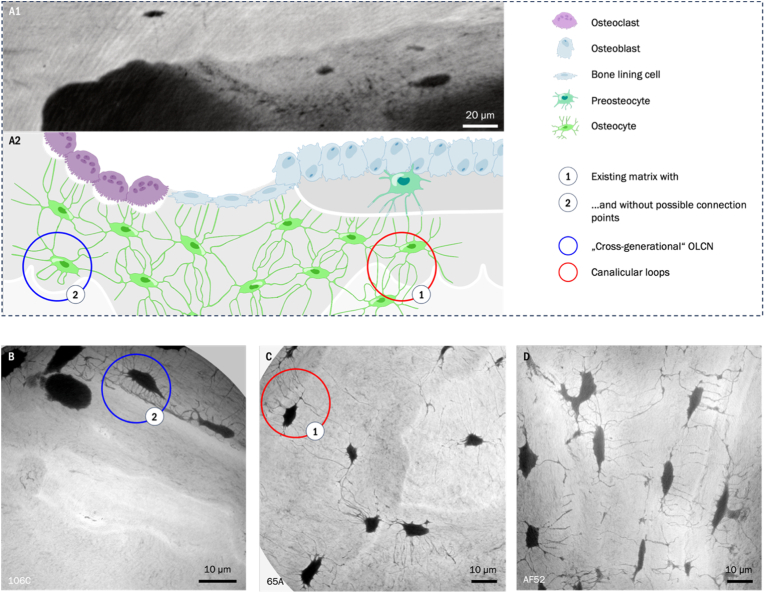
A1) Tomographic nano-CT image of bone remodeling in a BMU; A2) Schematic visualization of the proposed model of bone remodeling, highlighted with circles are the subprocesses and related canalicular behavior in dependency of local tissue/network conditions: development of an integrated network across regions of different local tissue age, canalicular loops in locations where no connections can be formed. B) - D) show additional minimum intensity projections (over 10 μm) of tissue regions showing the individual considered scenarios with different degrees of OLCN integration across cement lines.

Our findings support the existence of ‘cross-generational’ canalicular connections - structural links between osteocyte lacunae located in bone regions formed during different remodeling cycles (Fig. 7).

Reports on such connections have long been contradictory. While both early and more recent studies describe canalicular continuity across remodeling cycles [9,18,31], others considered them exceptions [17] or impossible due the supposed impermeability of cement lines [16]. Recent research consistently demonstrates their presence. Milovanovic et al. [9] describe canalicular continuity between osteonal and interstitial regions, emphasizing its role in maintaining mechano-sensation and nutrient transport. In line with these findings, our data reveals canalicular connections between interstitial and osteonal regions, and between adjacent osteons within all analyzed sample types.

A structural prerequisite for the formation of such connections is the presence of suitable contact points. The described orthogonal orientation of canaliculi towards the resorption interface maximizes the number of potential connection sites. This orientation reflects the generally normal orientation of canaliculi towards bone lamellae; the lamellar morphology in turn guides osteoclasts [32]. This implies that cell dendrites adjacent to resorption fronts must be able to survive the acidic microenvironment of matrix degradation [33]. Temporary retraction and later re-extension [34] can enable the establishment of connections during subsequent formation.

Beyond that, we identified canalicular loops near cement lines within the younger remodeling region. We interpret these as results from failed attempts at interconnection. This is supported by their exclusive occurrence directly toward cement lines, their increased appearance neighboring regions of low network density, and their absence when potential connections points are present. The analysis of the number of connections per lacuna and the canalicular densities of the respective neighboring remodeling regions underscores the strong dependency of the network integration on regional network density. Via the establishment of a significant decrease of canalicular porosity with the degree of mineralization, a reduction of network integration can be linked to the increase of local tissue age. The reported correlation of local tissue age and network density is in accordance with recent work by Jones et al. [35].

The presented data and observations convincingly show the network's ability and affinity to integrate across remodeling regions. The analysis presents local tissue conditions as the most influential factor to network integration. Finally, we observe both connecting and looping canalicular patterns in close proximity, suggesting that the mechanism underlying the network integration is highly local (Fig. 7).

The analysis of patterns of the canalicular architecture at the interface between different remodeling regions allows speculation on mechanisms involved in the network formation and integration. We propose a combination of an initial explorative mode and a responsive formation mode, depending on the local regulating environment. Initial extension of dendrites would occur stochastically. While within reach of local cues asymmetrically stimulating dendrite growth, the second mode would apply to initiate directed growth and the formation of connections.

This requires two aspects to be considered: the ability of the existing network or tissue to transmit directing cues and the ability of the newly forming network to shape its architecture in response. Potential cues relevant for initiating directed network integration can be physical and biological. Embedded osteocytes experience fluid flow induced mechanical stimulation. As the cell body of the osteocyte is known to orient in response to load [34], the outgrowth and orientation of cell processes is also driven by the activation of mechano-sensors [[36], [37]]. Both lacunae and canaliculi influence matrix strains [38]. Thus, created strain fields might play a role in determining dendrite development and canalicular network structure at remodeling interfaces. Cellular signaling by osteocytes, fundamental to the homeostasis of bone tissue and its dynamics [39], has also been shown to be crucial for network formation. Pathways such as prostaglandin E2 (PGE2) and Wnt/β-Catenin regulate dendrite outgrowth and osteocyte connectivity [4,5]. The connection between individual osteocyte dendrites (gap junction channel) heavily relies on connexins, particularly connexin 43 (Cx43). Cx 43 also mediates intercellular communication along these channels [40]. These signaling processes may stimulate forming dendrites locally and act as short range guides for the formation of ‘cross-generational’ connections.

Within the newly forming bone, tempo-spatial specifics of its structure and mineralization allow some flexibility in the arrangement of cell dendrites and canaliculi. The collagen lamellae of the bone serve as a general template for the cellular network architecture [32]. Canaliculi are confined to the disordered matrix phase between collagen lamellae, which mineralizes after the ordered phase [41,42], and formed during early mineralization [43]. Furthermore, active remodeling of the surrounding mineral matrix by osteocytes and cell processes (peri-lacunar/-canalicular remodeling), potentially achieved by digestion of the matrix [44,45], could enable adaptations of the canalicular orientation to establish connections. Thereby both forming and pre-existing tissue (matrix and cellular network) can facilitate and foster the ‘cross-generational’ integration of the OLCN.

An integrated network is likely to serve several functions. First, it enhances OLCN redundancy and thereby resilience, analogous to canalicular junctions [46] and clusters [10]. Second, it enables efficient mechanical signal transmission and biochemical communication across distinct remodeling units [12]. Third, these connections can contribute to more effective secondary mineralization, as the OLCN facilitates both the transport and release of mineral precursors and inhibitors [8,12,22,45]. The reported positive correlation between canalicular density and local calcium content [47] and the privileged access of the OLCN to the mineralized extra-cellular matrix (ECM) [48] supports their contribution to mineral regulation.

We therefore suggest that ‘cross-generational’ connections play a bi-directional role: maintaining the viability of pre-existing bone while enhancing integration and mineralization efficiency of newly formed tissue. By ensuring continuous access to a shared transport and signaling network, these connections support osteocyte survival and matrix quality on both sides of the cement line. The systematic formation of an integrated network, strongly influenced by its local environment, thus represents a fundamental strategy to support bone homeostasis and structural integrity.

### 3.2 Potential implications

The proposed model of a resilient ‘cross-generational’ osteocyte lacunar-canalicular network ties in with the current understanding of bone remodeling and maturation. The additional level of connectivity within the network will be relevant in various considerations regarding tissue integrity, a central goal in bone biology and regeneration.

In the context of age- or disease-related decline of bone quality the density and architecture of the OLCN has been recognized as a key factor [49]. A decline in canalicular porosity impairs cellular supply routes, rendering the bone matrix more susceptible to microdamage such as crack formation. This applies to ‘cross-generational’ connectivity on two scales: Age-related, reduced network density [9,49] will consequentially entail a reduced number of connections, additionally, reduced remodeling activity and increased mineral contents of the matrix may lead to a decline in possible locations of connections.

Moreover, the concept of an integrated, cross-generational network may open new avenues for regenerative strategies and bone tissue engineering. Large bone lesions due to e.g. severe bone fractures or cancer often require bone grafts. Adequate supply of the tissue is crucial for the integration of implants, native as well as synthetic. While macro-porosity has been widely acknowledged as essential for graft integration [50], the nano-scale OLCN, despite being the densest supply network in mineralized bone, has rarely been considered. With at least 50 % of the mineralized matrix lying within 1.5–2.1 μm of an OLCN structure [10], this network is uniquely capable of supporting metabolic exchange and integration, even in areas remote from vascular channels.

A consideration of the ‘cross-generational’ network will therefore be highly relevant in bone regeneration and in the context of conditions marked by compromised bone quality, such as osteoporosis.

### 3.3 Limitations

While the presented data robustly confirm the presence of canalicular connections across cement lines and loops in absence of connection points, CT imaging does not resolve the cellular processes within these structures. It remains possible that osteocyte processes retract within canaliculi [34,51], leaving them structurally intact but functionally disconnected. Direct confirmation of connectivity of the osteocyte dendrites across cement lines will require complementary methodologies such as fluorescence microscopy, histological staining, or live-cell imaging. Other authors, however, provide evidence on connections across cement lines based on biological imaging [52]. Additionally, every existing canaliculus did at one point house a functional osteocyte dendrite. Even if a connection might not be intact anymore it was formed in remodeling, which means the analysis holds true at least for a previous timepoint in the tissue graduation process. It should also be mentioned that the OLCN architecture changes with age and local tissue age. Continuous mineralization as well as pruning of canaliculi [53] entail a reduction of canaliculi. This potentially leads to an under-representation in connection numbers.

This study describes and interprets local canalicular patterns and derives the formation of ‘cross-generational’ connections as a systemic mechanism, based on their occurrence independent of sample parameters (such as age, sex, or pathology). Trends regarding different sample groups are not drawn. This decision is based on the heterogeneity of bone tissue on the micro- and nanoscale, the field-of-view (FOV) of the analyzed SR nano-CT scans, and the local character of the observed network features. Given an average scan size correlating to 0.0025 mm^3^ and an approximation of 20000 lacunae per mm^3^ [6,7], one tomographic nano dataset only captures around 50 individual lacunae. The dynamic nature of bone tissue implies a strong heterogeneity. Accordingly, even in healthy tissue numerous regions featuring a high degree of mineralization and decreased network density will be found. In consequence a randomly selected ROI of approximately 50 lacunae is insufficient to represent the condition of the entire sample or bone. The reoccurrence of the described patterns across the wide range of included samples, on the other hand, allows to assume a systematic mechanism, instead of an effect of a specific e.g. pathological condition.

Lastly, our quantitative analysis is constrained by the difficulties in segmentation and limited to datasets with sufficiently high image quality. Despite the resolution being high compared to other imaging techniques, it is still low in comparison to canalicular diameters resulting in partial volume effects inside the canalicular pores. Automated segmentation of the canalicular network in 3D therefore remains difficult. This study employes minimum intensity projections of 2D slices aiding the segmentation of the network. Projections entail potential errors due to the overlay of information, which must be taken into account in the assessment of the data. It is therefore important to note that the characterization of patterns relies in assessment of the 3D data, only the calculations are based on projections. Regarding the porosity assessment projected porosities are far higher than values based on calculations on the 3D data set would be. Thus, the reported values do not represent the actual regional porosities but rather a degree of porosity or network density, comparable between different regions. However, the overlay of individual canaliculi in a projection will lead to an underrepresentation of their number in the results. Thus, the here presented data can be assumed to be a conservative report of canalicular porosity and connections. In the future machine learning-based segmentation tools will significantly improve the 3D segmentation of the canalicular network and allow an even more realistic assessment of the networks complexity.

## 4 Conclusion

Revisiting the initially posed question, we support the existence of an integrated network across remodeling regions. We propose canalicular connections between regions of different remodeling cycles to be the result of a systemic mechanism. Based on the analysis of canalicular architecture patterns at remodeling interfaces we suggest a directed, responsive formation mechanism aimed at establishing network integration. This would contribute to tissue resilience, metabolic exchange, and structural integration. The presented analysis of canalicular network density and connections in correlation with local tissue conditions supports the hypothesis of the underlying integration mechanism, dependent on the local regulating environment. In this context, OLCN architecture patterns are introduced as indicators of functional intent in network development.

The proposed mechanism and the concept of a systematically integrated OLCN enhance our understanding of how bone maintains its functionality across cycles of resorption and formation. It includes new directions for interpreting transport dynamics, osteocyte survival, and network-driven mineralization processes. These insights lay the groundwork for future studies investigating how the spatial and temporal organization of the OLCN contributes to bone health—and how it might be targeted or restored in pathological conditions.

Ultimately, this study contributes to an evolving view of the OLCN as a highly dynamic, complex system whose roles in homeostasis, remodeling, and repair are broader and more sophisticated than previously appreciated.

## 5 Experimental section/methods

*Samples*: Bone samples were retrieved from consented human donors. This study includes 49 individual samples of femur, fibula, alveolar bone, and bone of the iliac crest and the fibula transplanted to alveolar sites from a total of 34 human donors (Table 1*)*. All included samples as well as age, sex, and health status of the donors are listed in the supplementary information in Table SI1. Details on samples measured during MD 672 have previously been reported [54]. Ethical approval for bone samples scanned during MD1363 and MD1405 was granted by the Charité's ethics committee (EA1/338/21).

**Table 1:** Number of samples from each of the included anatomical sites.

Anatomical site	Number of samples
Femur	7
Fibula	7
Alveolar	21
Iliac crest, transplanted	4
Fibula, transplanted	9

Total number	49

*Data Collection*: After excluding all scans not capturing remodeling events the total number of included tomographic data sets adds up to 85 scans.

The included synchrotron X-ray nano-CT data were collected during three different experiments at the European Synchrotron Radiation Facility (ESRF, Grenoble, France). MD672 was carried out at beamline ID22NI and experimental details have been previously reported [54]. MD1363 and MD1405 were carried out at beamline ID16B. During the experiment MD1363 the respective samples were scanned using a quasi-monochromatic focused beam (ΔE/E = 10^−2^) with an energy of 29.6 keV and 8.45 x 10^10^ photons/s incoming beam flux. In the first experiment set-up (set-up 1) a PCO edge 5.5 (scintillator LSO17) camera with 2560 x 2160 pixels image size was used as detector at 754 mm from the X-ray beam focus. The measurements were conducted following the propagation-based approach, scanning at 4 different focus-to-sample distances (58.355 mm, 59.355 mm, 63.355 mm, 73.355 mm) and moving the rotation axis off-center of the 2D projection to increase the FOV. At each distance, over 4000 images were aquired with a exposure time of 200 ms.

For MD1363 identical beam parameters where set. Scanning the samples BF32A, BF34A, KF25, and 6ARF, only a Germanium filter was used and the acquisition time reduced to 30 ms (set-up1). To further reduce beam exposure on the sample the set-up was changed during the course of the experiment (set-up 2) to a more efficient camera - a PCO edge 4.2 (scintillator LSO30) with 2048 x 2048 pixels image size. The scanning approach remained the same (with adjusted focus-to-sample distances of 54.572 mm, 55.572 mm, 59.572 mm, 69.572 mm). Here, at each distance 3788 projections were recorded over a total range of 360° with the rotation axis off center. The exposure time was set to 90 ms. For each sample, 1 or 2 single-distance (overview) scans were collected with an isotropic voxel size of 241 nm or 258 nm. Several regions of interest were then chosen to collect nano-CT scans with a voxel size of 50 nm. Due to the coherence of the synchrotron source, the intensity of the recorded radiograph includes phase contrast. To be serviceable for tomographic imaging, phase retrieval was performed using standard ESRF-developed holotomography codes [55,56]to extract the phase shift projections using an iterative algorithm based on the approach of Paganin for single-distance phase retrieval (δ/β = 325). The retrieved phase maps (3448 × 3448 pixels) are linearly related to mass density, which correlates to the degree of tissue mineralization [27]. The maps were used as input to a tomographic reconstruction algorithm based on filtered back projection (PyHST, ESRF, Grenoble, Fr) [57]. The 3D output was stacks of 2160 2D grayscale cross sections of 3448 x 3448 pixels. These result in a cylindrical volume of interest (VOI) with a diameter 830.968 μm or 889.584 μm and height of 520.56 μm or 557.28 μm for the respective voxel sizes of the oversize scans and a diameter of 172.4 μm and a height of 108.0 μm for the 50 nm scans.

During MD1405 the experimental set-up of MD1363 was followed and adjusted. Samples were again scanned using a quasi-monochromatic focused beam (ΔE/E = 10^−2^) with an energy of 29.6 keV. The PCO edge 5.5 camera was used but with another scintillator (LSO30). Due to a lower incoming beam flux (6.3 x 10^10^ photons/s) the exposure time was changed to 300 m s and the number of projections to 2003. The voxel sizes were 240 nm for the overview and 50 nm for the high-resolution scans. The reconstruction of the data followed the approach described for MD1363 yielding in stacks of 2160 2D grayscale cross sections of 2560 x 2560 pixels (and VOIs with a diameter of 614.4 μm and height of 518.4 μm for the overview scans and 128.0 μm and 108.0 μm for the nano scans). The most relevant scanning parameters are summarized in Table 2.

**Table 2:** Overview of SR nano-CT scanning parameters applied during the included experiments.

Experiment	Beamline	Energy [keV]	Flux [photons s^−1^]	Detector	Distance to source [mm]	Projections []	Exposure time [ms]	Paganin length, δ/β
MD672	ID22NI	29.6	8.45 x 10^10^	PCO edge 5.5 (LSO17)	754	>4000	200	199
MD1363								
set-up 1	ID16B	29.6	8.45 x 10^10^	PCO edge 5.5 (LSO17)	705	3788	30	325
set-up 2		29.6	8.45 x 10^10^	PCO edge 4.2 (LSO30)	705	3788	90	325
MD1405	ID16B	29.6	6.3 x 10^10^	PCO edge 5.5 (LSO30)	705	2003	300	325

*Data Selection:* All 50 nm data sets from the above mentioned beamtimes were included in an initial screening. This was performed to identify remodeling events and interfaces between different remodeling regions within the scans. All data set containing at least one remodeling event (resorption, formation or both in proximity) or a cement line were selected and included in the study together with the respective overview scans (voxel size: 240–250 nm). All included scans are listed in Tabel SI.1.

*Data Analysis*: After image reconstruction, processing of the data was performed using Fiji, an ImageJ distribution (NIH, Bethesda, MD, USA), Avizo (Avizo 9.7.0, Thermo Fischer Scientific Inc., Waltham, MA, USA), and IPSDK Explorer (Reactiv’IP Smart Image Processing, Grenoble, France). To guarantee comparability across samples only alveolar samples - including at least two distinct remodeling regions - from one beamtime (MD672) were included in the quantitative analysis. Tomographic image stacks were rotated and resliced such that the most prominent 3D cement line structure was parallel to the stack's z-axis.

1) Degree of mineralization: To assess the degree of mineralization for individual remodeling regions gray value analysis was performed on the transformed 3D tomographic image stack. In a first step the individual remodeling regions were manually labeled in individual slices and the regional mask created by interpolation of the manual labels (A). The accuracy of the interpolated regional masks was confirmed by matching them with the gray value data. A mask containing all low intensity elements (lacunae, canaliculi, vessel structures, and image noise) was created using an Otsu threshold [58] (B). Additionally, a lacunar mask was calculated based on an initial Otsu mask, followed by an opening of seven voxels to separate the individual elements, a connected component analysis, a consecutive size filtering to exclude elements below a 1500 voxel threshold, and a final binarization (C). Based on these initial masks the mean regional gray value of the mineralized matrix was assessed within each remodeling region. To do so, an erosion with a structuring element size of 50 pixel was performed on the regional mask to exclude potential edge effects in the data sets as well as the cement lines. Mask B was dilated by 10 pixels, mask C eroded by 10 pixels. To assign lacunae to their remodeling region mask C was multiplied with mask A. The mineral matrix was masked by subtraction of the dilated mask B from mask A. Mean gray values were calculated per region within the mineral mask as well as within the eroded lacunar mask. To account for low frequency artefacts of local tomographic imaging, mean gray value results of the mineral matrix were normalized to the lacunar results by region, allowing the assessment of intensity differences between the mineralized and non-mineralized phase [54]. Based on these values (normalized mean GV) the relative degree of mineralization was derived and compared across remodeling regions of each sample. Regions within each sample were ranked from one (youngest) to two or three (oldest) depending on the number of included remodeling regions. Regions of minimal size were excluded from consideration.
2) Projected canalicular porosity: To compare the canalicular porosity canaliculi were segmented in 15 individual minimum intensity projections over 130 slices (=6.5 μm) for each data set. Projections over the same slices were also created of the lacunar and the regional mask. The segmentation was performed applying a top hat segmentation algorithm with a structuring element size of 10 pixel followed by an Otsu thresholding. A connected component analysis was performed on the resulting mask to afterwards filter by size excluding components below 100 voxels defined as noise. The filtered mask was then re-binarized. To assign the network to the respective remodeling region the canalicular mask was multiplied with the projected regional mask. Both lacunar and canalicular mask were subtracted from the regional mask to create a regional mineral mask. Projected regional canalicular volume (pCan.V(i)) was calculated as the sum of canalicular pixel per region label. Projected mineral volume (pMin.V(i)) was calculated parallelly as the sum of all mineral pixel per region label. Both values were calculated for all projections (i) and projected canalicular porosity (Can.P) defined as follows:

$$pCan.P=\frac{\Sigma pCan.V\left(i\right)}{\Sigma pMin.V\left(i\right)}$$

Projected canalicular porosity was assessed over relative degree of mineralization as well as over normalized mean GV per remodeling region for each sample.

3) Canalicular connections: The number of canalicular connections between remodeling regions per lacuna was assessed based on the previously described masks. A total of 24 lacunae were included based on the following criteria: in proximity to the cement line, within the locally younger remodeling region, fully included in the scan. For each of the selected lacunae new projections were created from all slices including the lacuna, this was done separately over identical i-range for the lacunar, canalicular, and regional mask. From the lacunar projection all other lacunae were excluded, the relevant lacuna was then added to the canalicular mask. With this new mask, a connected component analysis was performed and the largest components kept, which included the lacuna and all canaliculi directly connected to it. In the regional mask the interface to the locally older region was segmented by dilating the label of the older region by 3 pixel and subtracting the original label. The canalicular cluster was intersected with the segmented interface to the older region. Individual elements of this intersection within 25 μm from the lacuna were counted as the number of canalicular connections between remodeling regions (*N.Conn*). The number of canalicular connections per lacuna was assessed over normalized mean GV of the region of the lacuna and the neighboring (older) region.

**Fig. 8:**
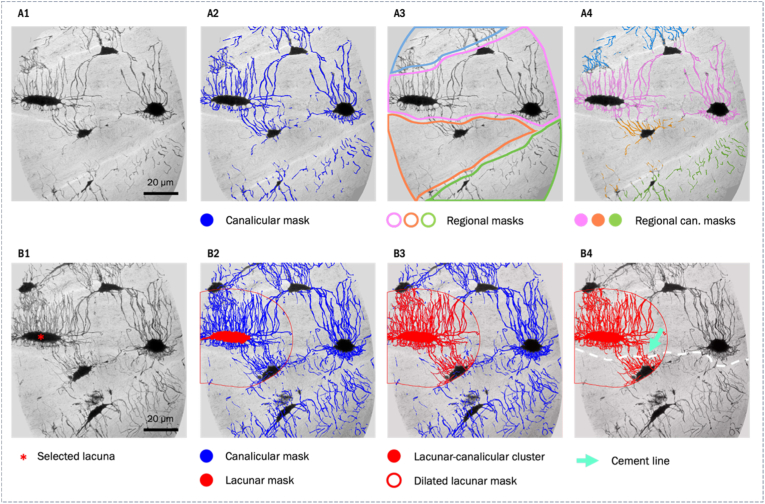
A) Shows the segmentation workflow used for the calculation of projected canalicular porosity values (A1) minimum intensity projection over 130 slices – A2) canalicular mask – A3) regional mask – A4) regional canalicular mask); B) Shows the segmentation workflow based for the counting of canaliculi per lacuna crossing the cement line (B1) minimum intensity projection over lacunar height – B2) canalicular mask – B3) canalicular mask components connected to analyzed lacuna – B4) connected component and cement line mask).

An illustration summarizing points 1-3) is shown in Fig. 8.

Data sets included in the quantitative analysis, as well as results are accessible under the following https://doi.org/10.5281/zenodo.17909733.

*Statistical Analysis*. The analysis of median projected canalicular porosity over relative degree of mineralization of remodeling regions was statistically assessed using student's *t*-test after analysis of correlation. The median over youngest region group was compared to the combined median of the relatively older groups. The statistical analysis of the distribution of canalicular connections per lacuna over normalized mean GV of locally younger and older remodeling region was performed based on a Pearson correlation and a consecutive student's *t*-test. Results were considered statistically significant for p-values p < 0.05.

## CRediT authorship contribution statement

**Sophie Anuth:** Writing – review & editing, Writing – original draft, Visualization, Validation, Methodology, Investigation, Formal analysis, Data curation, Conceptualization. **Emely Bortel:** Writing – review & editing, Writing – original draft, Methodology, Investigation, Data curation. **Julie Villanova:** Writing – review & editing, Methodology, Investigation. **Jussi-Petteri Suuronen:** Writing – review & editing, Methodology, Investigation, Formal analysis. **Sven Geissler:** Writing – review & editing, Writing – original draft, Investigation. **Amaia Cipitria:** Writing – review & editing, Supervision, Investigation. **Peter Fratzl:** Writing – review & editing, Validation, Methodology. **Tobias Fretwurst:** Writing – review & editing, Investigation. **Katja Nelson:** Writing – review & editing, Supervision, Methodology, Investigation, Funding acquisition. **Susanne Nahles:** Writing – review & editing, Supervision, Methodology, Investigation. **Bernhard Hesse:** Writing – review & editing, Writing – original draft, Visualization, Validation, Supervision, Software, Resources, Project administration, Methodology, Investigation, Funding acquisition, Formal analysis, Data curation, Conceptualization.

## Declaration of competing interest

The authors declare that they have no known competing financial interests or personal relationships that could have appeared to influence the work reported in this paper.

## Data Availability

Data sets included in the quantitative analysis, as well as results are accessible under the following DOI: 10.5281/zenodo.17909733
