# Supplementary material for: Nano-scale evidence for osteocyte network integration across bone remodeling interfaces in human bone revealed by synchrotron nanoCT
Source: Mater Today Bio. 2026 Jan 16;37:102813. doi: 10.1016/j.mtbio.2026.102813 (PMC12859465; doi:10.1016/j.mtbio.2026.102813)
Supplement: Multimedia component 1 [file mmc1.docx]

**Supplementary information:**

**Nano-scale evidence for osteocyte network integration across bone remodeling interfaces in human bone revealed by synchrotron nanoCT**

*Sophie Anuth^1,2,3^, Emely Bortel^1^, Julie Villanova^4^,* *Jussi-Petteri Suuronen^1^, Sven Geissler^5^, Amaia Cipitria^2, 6^, Peter Fratzl^7^, Tobias Fretwurst^8^, Katja Nelson^8^, Susanne Nahles^9^, Bernhard Hesse^1,^**

^1^ Xploraytion GmbH, Invalidenstrasse 34, 10115 Berlin, Germany.

^2^ Group of Bioengineering in Regeneration and Cancer, Biogipuzkoa Health Research Institute, San Sebastián, Spain.

^3^ Department of Cell Biology and Histology, Faculty of Medicine and Nursing, University of the Basque Country UPV/EHU, Leioa, Spain.

^4^ ESRF – European Synchrotron Facility, Avenue des Martyrs 71, 38043 Grenoble, France.

^5^ Berlin Institute of Health Center for Regenerative Therapies, Charité - Universitätsmedizin Berlin, Berlin, Germany.

^6^ IKERBASQUE, Basque Foundation for Science, Bilbao, Spain.

^7^ Department of Biomaterials, Max Planck Institute of Colloids and Interfaces, Max Planck Society, Am Mühlenberg 1 OT Golm, 14476 Potsdam, Germany.

^8^ Department of Oral and Craniomaxillofacial Surgery/Translational Implantology, Center for Dental Medicine, Medical Center, Faculty of Medicine, University of Freiburg, Hugstetter Straße 55, Freiburg 79106, Germany

^9^ Department of Craniomaxillofacial Surgery, Charité, CVK, Augustenburger Platz 1, Berlin 13353, Germany.

**Table SI.1**: List of all included samples scanned at 50 nm pixel size sorted by the respective experiments. (Scans were selected for showing at least one (re)modeling event.)

| Experiment | Sample | Scan | Site | Sex | Age | Comment | Conn. | Loop |
| --- | --- | --- | --- | --- | --- | --- | --- | --- |
| MD672 | fem_1LF70 | 1 | Femur | F | 70 |  |  | X |
|  | fem_2RM60 | 1 | Femur | M | 60 |  | X | X |
|  | fem_5RF68AM | 1 | Femur | F | 68 |  | X |  |
|  | fem_11LF87 | 1 | Femur | F | 87 |  |  | X |
|  | fem_15RF66 | 1 | Femur | F | 66 |  | X | X |
|  | FEM11LF68 | 1 | Femur | F | 68 |  |  |  |
|  | FEM1LM71 | 1 | Femur | M | 71 |  |  | X |
|  | jaw_1mk4 | 1 | Alveolar | M | 84 | BRONJ (10 y), Prostate-carcinoma | X |  |
|  |  | 2 |  |  |  |  | X | X |
|  | jaw_1wk4 | 1 | Alveolar | F | 75 | BRONJ (13 m), Mammary-carcinoma | X | X |
|  | jaw_1wk5b | 1 | Alveolar | F | 70 | BRONJ (16 m), Plasmacytom | X | X |
|  | jaw_2mg4 | 1 | Alveolar | M | 42 | - | X | X |
|  | jaw_2wg3B | 1 | Alveolar | F | 40 |  | X | X |
|  | jaw_2wg4 | 1 | Alveolar | F | 47 |  | X | X |
|  | jaw_2wg5 | 1 | Alveolar | F | 42 |  | X | X |
|  | jaw_3wk1a | 1 | Alveolar | F | 72 | BRONJ (10 y), Osteoporosis | X | X |
|  | jaw_2wg1 | 1 | Alveolar | F | 68 | - | X |  |
| MD1363 | BF34A | 1 | Alveolar/iliac crest | F | 72 | Transplanted |  | X |
| set-up 1 |  | 2 |  |  |  |  | X |  |
|  |  | 3 |  |  |  |  |  | X |
|  |  | 4 |  |  |  |  |  | X |
|  | 6ARF | 1 | Alveolar | F | 69 | Radiation | X |  |
|  |  | 2 |  |  |  |  | X |  |
|  |  | 3 |  |  |  |  | X | X |
| set-up 2 | 9ARF | 1 | Alveolar | M | 74 | Radiation |  | X |
|  |  | 2 |  |  |  |  |  | X |
|  | KF44 | 1 | Alveolar | M | 63 | Radiation | X | X |
|  |  | 2 |  |  |  |  | X | X |
|  | SC42 | 1 | Alveolar |  |  | Body donor | X |  |
|  |  | 2 |  |  |  |  | X | X |
|  |  | 3 |  |  |  |  | X | X |
|  |  | 4 |  |  |  |  | X |  |
|  |  | 5 |  |  |  |  | X | X |
|  |  | 6 |  |  |  |  | X |  |
|  | 130C | 1 | Alveolar | M | 60 | Free fibula flab | X | X |
|  | 130Dx | 1 | Alveolar/ fibula |  |  |  | X |  |
|  |  | 2 |  |  |  |  | X | X |
|  |  | 3 |  |  |  |  | X | X |
|  |  | 4 |  |  |  |  | X | X |
|  |  | 5 |  |  |  |  | X |  |
|  | 130F | 1 | Fibula | M |  |  |  |  |
|  |  | 2 |  |  |  |  | X | X |
|  | 116C | 1 | Alveolar | M | 64 | Free fibula flab | X | X |
|  |  | 2 |  |  |  |  | X | X |
|  |  | 3 |  |  |  |  | X | X |
|  |  | 4 |  |  |  |  |  |  |
|  |  | 5 |  |  |  |  | X | X |
|  |  | 6 |  |  |  |  | X | X |
|  |  | 7 |  |  |  |  | X | X |
|  | 116B | 1 | Fibula |  |  |  | X |  |
|  | 116A | 1 | Alveolar/fibula |  |  |  |  | X |
|  |  | 2 |  |  |  |  |  | X |
|  | AF52 | 1 | Alveolar | F | 68 | Radiation | X | X |
|  |  | 2 |  |  |  |  | X | X |
|  |  | 3 |  |  |  |  | X | X |
|  | BF39A | 1 | Alveolar | F | 66 | Transplanted |  |  |
|  | BF40A | 1 | Alveolar/iliac crest | F | 66 | Transplanted | X | X |
|  |  | 2 |  |  |  |  | X | X |
|  |  | 3 |  |  |  |  | X | X |
|  |  | 4 |  |  |  |  | X | X |
| MD1405 | 65A | 1 | Alveolar | F | 57 | Free Fibula Flab, radiation | X | X |
|  | 65B | 1 | Fibula |  |  |  |  | X |
|  | 65C | 1 | Alveolar/fibula |  |  |  |  | X |
|  |  | 2 |  |  |  |  |  | X |
|  | 47C | 1 | Alveolar/fibula | M | 72 | Free Fibula Flab, radiation |  | X |
|  | 47BII | 1 | Fibula |  |  |  | X | X |
|  | 60A | 1 | Alveolar | M | 79 | Free Fibula Flab |  |  |
|  | 60B | 1 | Fibula |  |  |  |  |  |
|  | 60C | 1 | Alveolar/fibula |  |  |  | X |  |
|  | 70B | 1 | Fibula | M | 53 | Free Fibula Flab, radiation |  | X |
|  | 70C | 1 | Alveolar/fibula |  |  |  | X | X |
|  |  | 2 |  |  |  |  | X | X |
|  |  | 3 |  |  |  |  | X | X |
|  | 44F | 1 | Alveolar/fibula | M | 73 | Free fibula flab | X | X |
|  | 44C | 1 | Fibula |  |  |  | X | X |
|  |  | 2 |  |  |  |  |  | X |
|  | 106A | 1 | Alveolar | M | 71 | Free fibula flab | X | X |
|  | 106C | 1 | Alveolar/fibula |  |  |  |  | X |
|  |  | 2 |  |  |  |  | X | X |
|  | 106D | 1 | Fibula |  |  |  | X | X |
|  | BF32A | 1 | Alveolar/iliac crest | F | 59 | Transplanted |  | X |
|  | BF39A | 1 | Alveolar/iliac crest | F | 66 | Transplanted |  | X |
|  | 46A | 1 | Alveolar | F | 46 | Free fibula flab | X | X |
|  | 46C | 1 | Alveolar/fibula |  |  |  |  | X |
|  |  | 2 |  |  |  |  | X | X |

**Table SI.2**: Results of normalized mean GV and projected canalicular porosity per (re)modeling region and sample.

| Sample | Age | (Re)modeling Region -  Relative Local Tissue Age | Normalized Mean GV | Projected Canalicular Porosity Proj. (Can.V/Min.V) |
| --- | --- | --- | --- | --- |
| 2mg4 | 42 | 1 | 34113.01 | 0.0954 |
|  |  | 2 | 35121.10 | 0.0974 |
|  |  | 3 | 37507.66 | 0.0577 |
| 2wg1 | 68 | 1 | 32721.71 | 0.0820 |
|  |  | 2 | 32820.8 | 0.0996 |
| 2wg4 | 47 | 1 | 37607.91 | 0.1028 |
|  |  | 2 | 40310.91 | 0.0396 |
|  |  | 3 | 42097.91 | 0.0317 |
| 2wg5 | 42 | 1 | 38878.45 | 0.0891 |
|  |  | 2 | 39265.41 | 0.0929 |
|  |  | 3 | 39229.75 | 0.0593 |
| 1wk4 | 75 | 1 | 39079.91 | 0.0780 |
|  |  | 2 | 41749.24 | 0.0676 |
| 3wk1 | 72 | 1 | 26180.82 | 0.0717 |
|  |  | 2 | 27725.84 | 0.0341 |
|  |  | 3 | 31227.7 | 0.0654 |

**Table SI.3**: Results of number of total extending canaliculi and canaliculi crossing cement line towards neighboring (re)modeling region, as well as projected canalicular porosity of younger and neighboring, older region per analyzed lacuna (within locally younger regions).

| Sample | Lacuna | (Re)modeling Region -  Proj. Canalicular Porosity | Neighboring Region –  Proj. Canalicular Porosity | Extending Canaliculi | Connecting Canaliculi |
| --- | --- | --- | --- | --- | --- |
| 2mg4 | 1 | 0.1647 | 0.0971 | 27 | 16 |
|  | 2 | 0.1647 | 0.0971 | 37 | 7 |
|  | 3 | 0.1647 | 0.0971 | 44 | 13 |
|  | 4 | 0.1647 | 0.0971 | 16 | 11 |
|  | 5 | 0.1611 | 0.0971 | 35 | 2 |
|  | 6 | 0.1611 | 0.0971 | 29 | 15 |
|  | 7 | 0.1611 | 0.0971 | 65 | 17 |
| 2wg1 | 1 | 0.1697 | 0.1719 | 34 | 25 |
| 2wg4 | 1 | 0.2158 | 0.0913 | 33 | 3 |
|  | 2 | 0.2158 | 0.0913 | 39 | 0 |
|  | 3 | 0.2158 | 0.0913 | 45 | 7 |
|  | 4 | 0.2158 | 0.0913 | 31 | 0 |
|  | 5 | 0.2158 | 0.0913 | 28 | 4 |
|  | 6 | 0.0563 | 0.0913 | 19 | 0 |
|  | 7 | 0.0563 | 0.0913 | 19 | 10 |
| 2wg5 | 1 | 0.1144 | 0.1877 | 56 | 15 |
|  | 2 | 0.1144 | 0.1877 | 31 | 18 |
|  | 3 | 0.1144 | 0.1877 | 34 | 44 |
|  | 4 | 0.1144 | 0.1877 | 38 | 25 |
| 1wk4 | 1 | 0.1638 | 0.1495 | 41 | 6 |
|  | 2 | 0.1638 | 0.1495 | 31 | 0 |
|  | 3 | 0.1638 | 0.1495 | 52 | 24 |
|  | 4 | 0.1638 | 0.1495 | 42 | 12 |
| 3wk1 | 1 | 0.1271 | 0.0473 | 33 | 11 |
|  | 2 | 0.1271 | 0.0473 | 58 | 7 |
|  | 3 | 0.1271 | 0.0473 | 18 | 10 |
|  | 4 | 0.1184 | 0.0473 | 33 | 10 |


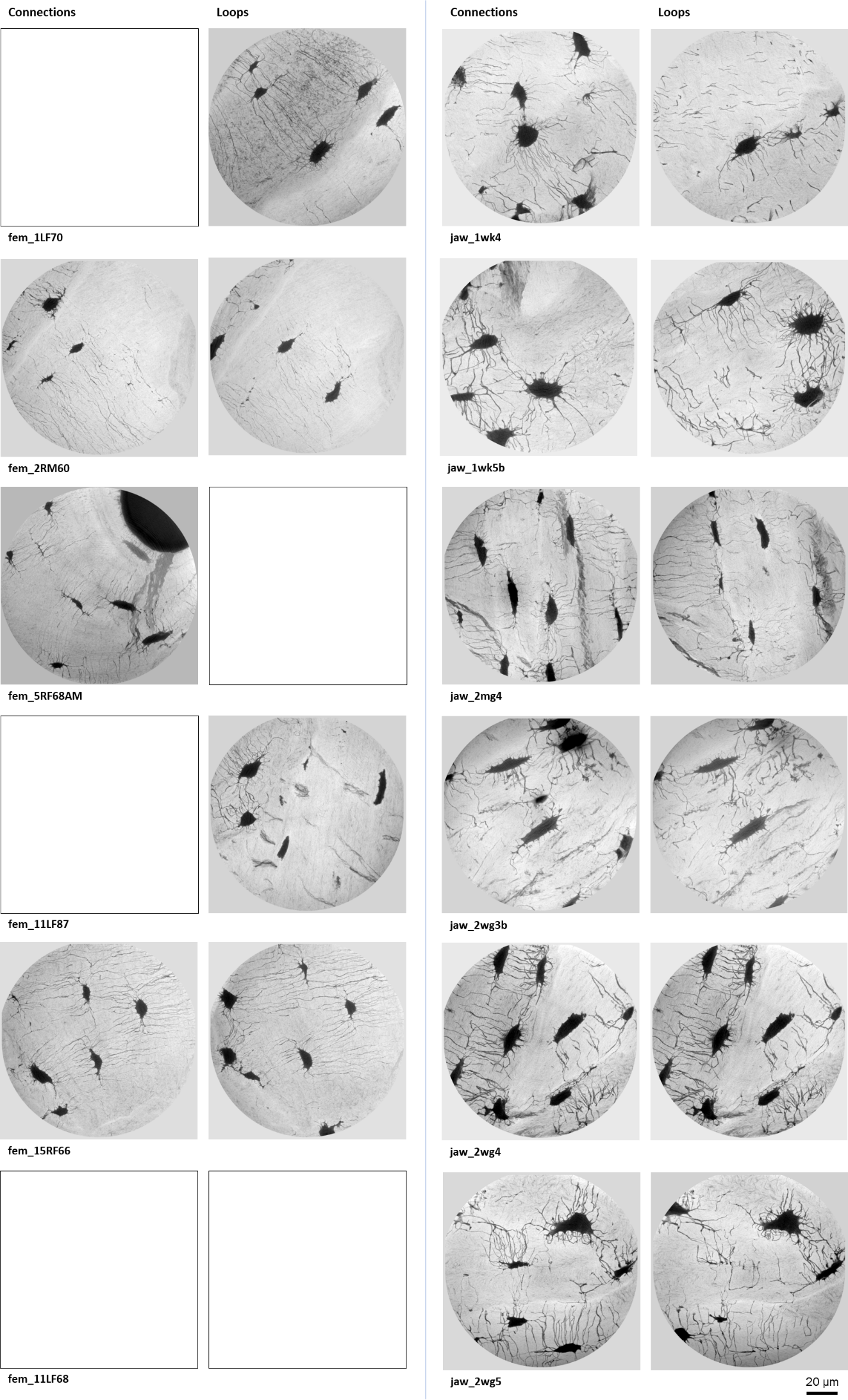


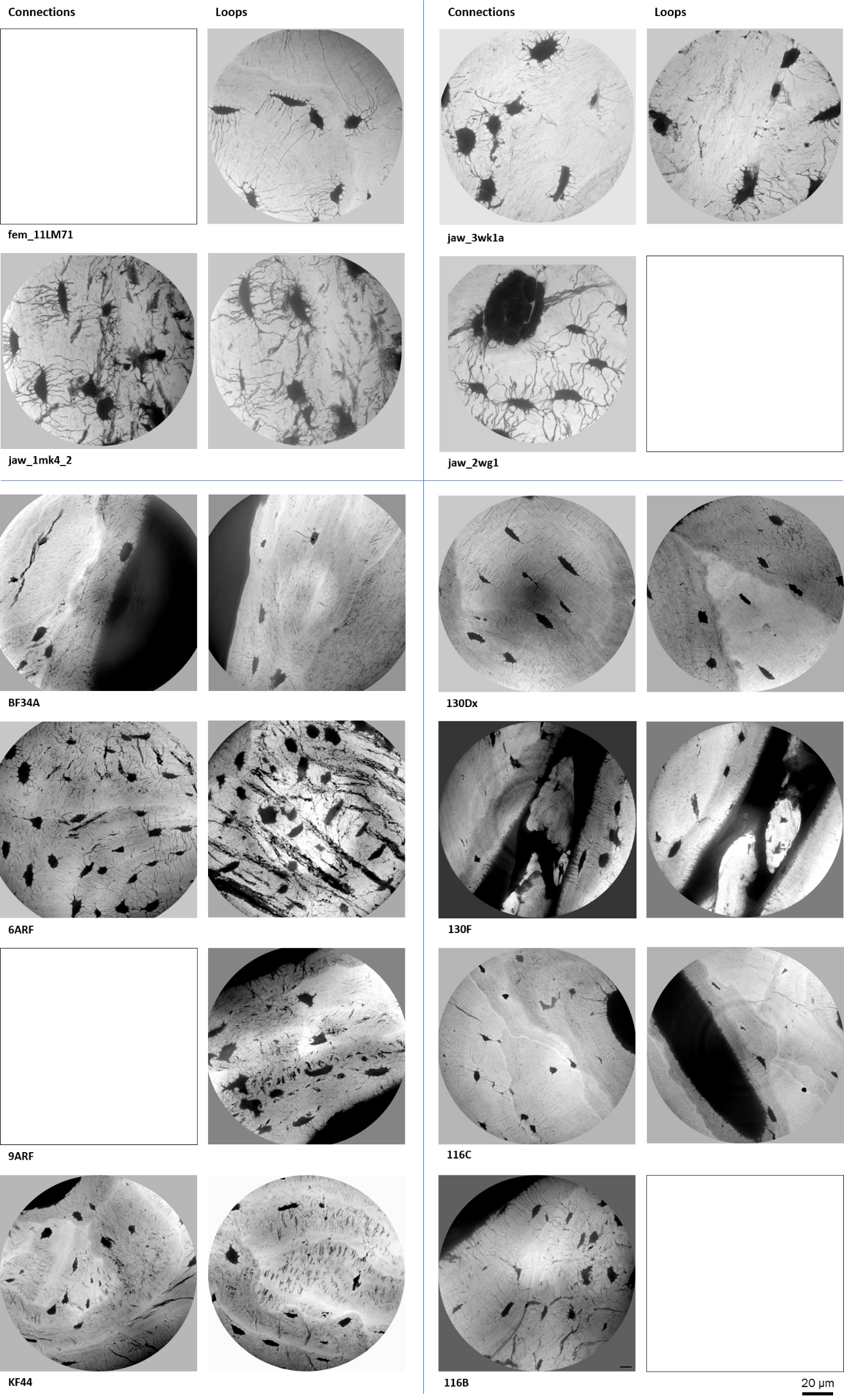


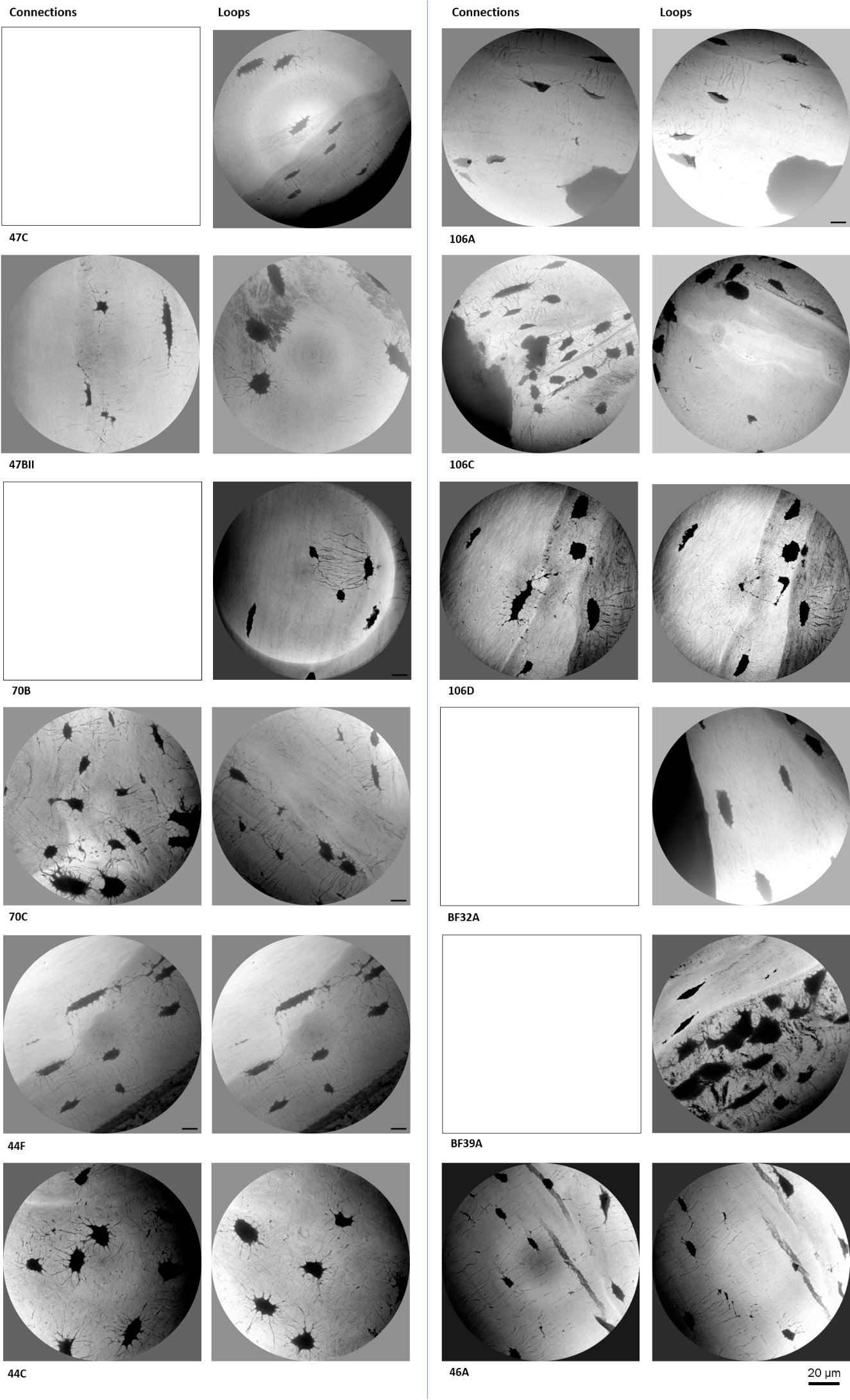

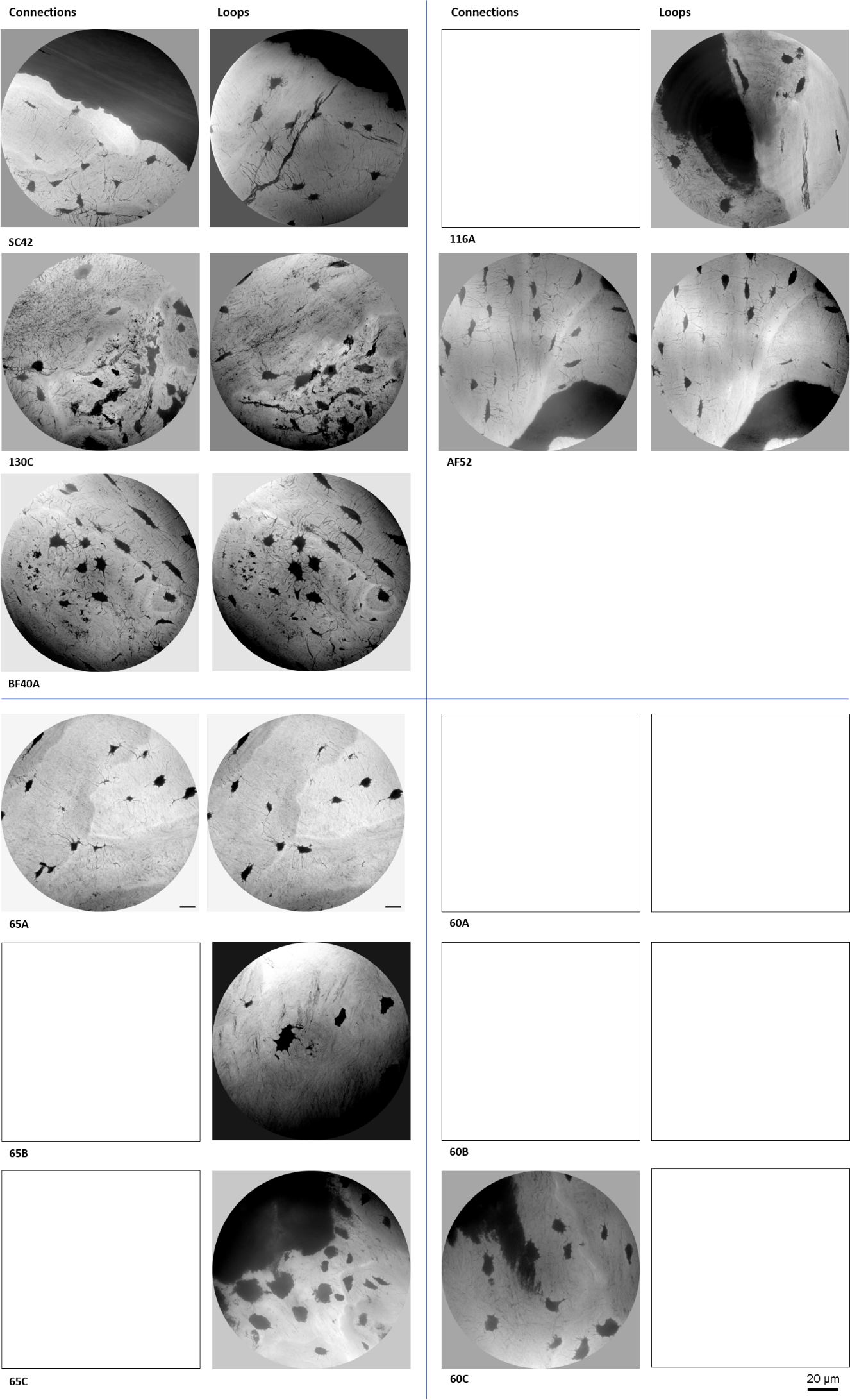

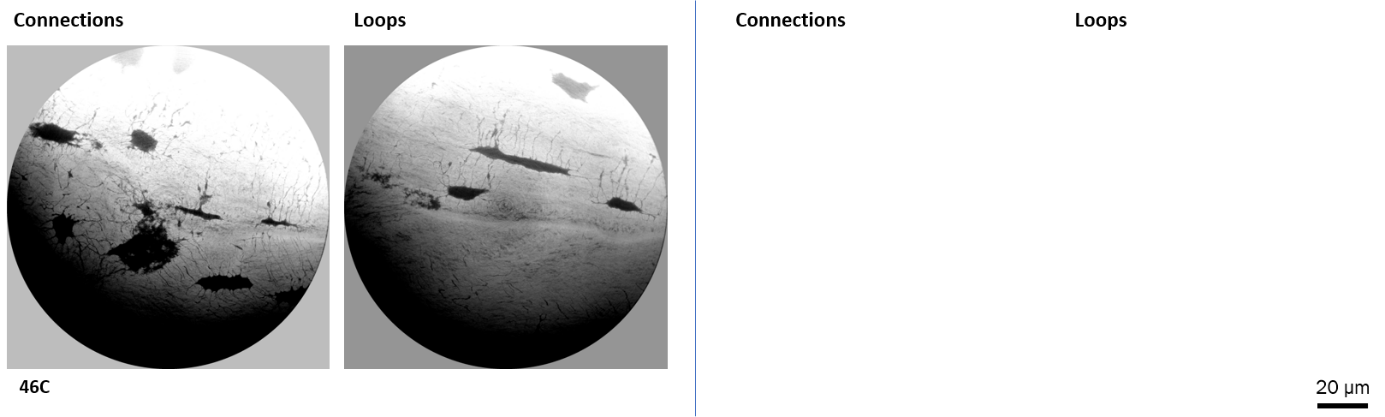


**Figure SI.1.**Minimum intensity projections over 10 µm showing connections and loops in one exemplary location per sample; empty frames indicated that the respective pattern could not be found in the corresponding data-sets. All canalicular patterns and the positions of cement lines were carefully assessed for each sample. (Cement lines are, depending on their angles towards the stack´s z-axis (projection direction), not always well identifiable in the projection images.)
